# Retrospective Analysis of Checkpoint Inhibitor Therapy-Associated Cases of Bullous Pemphigoid From Six German Dermatology Centers

**DOI:** 10.3389/fimmu.2020.588582

**Published:** 2021-02-23

**Authors:** Christian D. Sadik, Ewan A. Langan, Ralf Gutzmer, Maria Isabel Fleischer, Carmen Loquai, Lydia Reinhardt, Friedegund Meier, Daniela Göppner, Rudolf A. Herbst, Detlef Zillikens, Patrick Terheyden

**Affiliations:** ^1^Department of Dermatology, Allergy, and Venereology, University of Lübeck, Lübeck, Germany; ^2^Center for Research on Inflammation of the Skin (CRIS), University of Lübeck, Lübeck, Germany; ^3^Dermatological Sciences, University of Manchester, Manchester, United Kingdom; ^4^Skin Cancer Center Hannover, Department of Dermatology, Hannover Medical School, Hannover, Germany; ^5^Department of Dermatology, University Medical Center Mainz, Mainz, Germany; ^6^Department of Dermatology, University Hospital Dresden, Dresden, Germany; ^7^Department of Dermatology, University of Gießen, Gießen, Germany; ^8^Department of Dermatology, HELIOS Klinikum Erfurt, Erfurt, Germany

**Keywords:** checkpoint inhibitors, autoimmunity, pemphigoid disease, autoantibodies, pembrolizumab, nivolumab, PD-1 - PD-L1 axis, ipilimumab

## Abstract

Immune-related adverse events (irAEs) are a class-effect of checkpoint inhibitors (CIs). The development of a Bullous pemphigoid (BP)-like blistering disease, driven by autoantibodies against the hemidesmosomal protein BP180, is a potentially serious irAE whose incidence seems to be increasing. We therefore set out to characterize the clinical and (immuno)histopathological features and treatment responses of cases of BP which developed during or after CI therapy collated in six German tertiary referral centers between 2014 and 2018. We identified twelve cases of BP which emerged during and/or after CI therapy. The time interval between the initiation of CI therapy and the diagnosis of BP was 3–74 weeks (median: 23 weeks). Age at the time of diagnosis of BP varied between 62 and 80 years (median: 76 years). The clinical presentation of the patients was diverse but the severity was relatively mild when compared to that seen in most cases of spontaneous BP. Only four patients met all of the immunopathological criteria recommended in the European guidelines for the diagnosis of BP. Topical corticosteroid treatment was sufficient to achieve disease control in most patients. CI therapy could be continued in 8 out of 12 patients. In summary, our study indicates that cases of BP during or after CI therapy bear several peculiarities distinguishing them from spontaneous BP. Given the diversity of the clinical presentation of CI-induced BP the application of existing diagnostic algorithms developed for spontaneous BP can be utilized to uncover the frequency and features of CI-induced BP and to develop and optimize management algorithms.

## Background

Checkpoint inhibitors (CIs) constitute a new class of immunomodulatory drugs which block co-inhibitory signals on immune effector cells and result in the generation of potent T-cell mediated immune responses. Among the CIs which are currently licensed are monoclonal antibodies which target the cytotoxic T-lymphocyte-associated protein 4 (CTLA-4) or the programmed cell death protein 1 (PD-1) pathway. For example, the first-in-class drug ipilimumab inhibits CTLA-4, nivolumab and pembrolizumab inhibit PD-1, and atezolizumab and durvalumab target the PD-1 ligand PD-L1 (1). The advent of checkpoint inhibitors has revolutionized the treatment of solid cancers. Initially licensed for the treatment of metastatic melanoma, CIs are employed in an ever increasing number of cancer entities, including Merkel cell carcinoma, head and neck squamous cell carcinoma, renal cell carcinoma, non-small cell lung cancer, urothelial bladder cancer, hepatocellular carcinoma, and Hodgkin’s lymphoma (1).

Given that the CTLA-4 and PD-1 pathways play a central role in regulating cellular immune responses, removal of this control mechanism can result in both generalized and tissue-specific inflammation. These inflammatory responses are collectively termed “immune-related adverse events” (irAEs), and include, but are not limited to, dermatitis, colitis, hypophysitis, hepatitis, and nephritis (1). The skin is the most frequently affected organ, with up to 50% of patients receiving CIs developing skin-related irAEs.

The clinical presentation of cutaneous irAEs is remarkably diverse. Commonly presenting with a non-specific maculopapular rash accompanied by pruritus, they may also present with lichenoid, eczematous, granulomatous, lupus-like, or erythema multiforme-like skin changes and/or vitiligo (2).

Most intriguingly, there is a growing body of literature which reports the development of the antibody-driven autoimmune disease bullous pemphigoid (BP), the most common disease of the group of pemphigoid diseases, in the context of treatment with immune CI (2–8).

The hallmark of BP is the development of autoimmunity against type XVII collagen (BP180) (9). BP presents clinically with the development of widespread urticarial plaques (pre-bullous phase), which evolve into blisters and erosions (10, 11). However, non-bullous and atypical variants of BP also exist. In these variants, patients exhibit localized blister formation with minimal or absent inflammation of the surrounding skin. Additionally, BP may present with predominantly eczematous (including dyshidrosiform) and subacute prurigo-like skin changes (12). Indeed, BP may exist in the absence of skin changes, with the only symptom being widespread, intractable itch. Given the wide spectrum of clinical symptoms and signs in BP, the disease can neither be reliably diagnosed nor excluded based on the clinical presentation alone. The European Academy of Dermatology and Venereology (EADV) guidelines recommend that the diagnosis of BP should be based on both the clinical findings and (immuno)pathological investigations of the patient’s skin, both lesional and perilesional, and serum (12). Central to establishing the diagnosis of BP is the demonstration of linear depositions of autoantibodies, which are in most cases predominantly of the IgG immunoglobulin class, and/or of complement factor 3 (C3) at the dermal-epidermal junction (DEJ) of perilesional skin (12). As the deposition of autoantibodies and/or complement at the DEJ is the defining feature of all pemphigoid diseases and as the clinical features of BP may overlap with those of other pemphigoid diseases, the clear distinction of pemphigoid diseases requires the detection of autoantibodies in the serum and a determination of their antigen specificity. This is achieved by indirect immunofluorescence microscopy on NaCl-split human skin, which detects the presence of autoantibodies against proteins of the dermal-epidermal junction, and by ELISA biochip-based techniques, and/or immunoblot assays to pinpoint the antigen specificity of the autoantibodies. Serological examination alone is not sufficient to diagnose BP given that autoantibodies are reportedly undetectable in the serum of up to 20% of BP patients, but are present in the serum of 0.5% of healthy individuals (12, 13). Histopathologically, an early blister exhibits a subepidermal cleft combined with a dense dermal inflammatory infiltrate mainly consisting of eosinophils and neutrophils. However, the histopathological examination is also non-specific and is therefore not suitable alone to confirm the diagnosis (14).

The complexity and heterogeneity of both BP and cutaneous irAEs means that a comprehensive clinical, serological, and (immuno)histopathological work-up is central to correctly diagnosing suspected cases of BP during or after CI therapy. In the present study, we retrospectively profiled twelve cases of CI-associated BP diagnosed in six German Dermatology centres and contrasted their features with previously reported cases of both immune-checkpoint mediated and spontaneous BP.

## Methods

We systematically searched the clinical records of patients treated with CIs between 2014 and 2018 in six German Dermatology centres for the diagnosis of BP. The case notes of affected patients were then retrospectively analyzed to determine the salient clinical, histopathological, and immunopathological features. Histo- and immunopathological analyses were conducted by routine autoimmune and dermatohistopathology laboratories. Ethical approval was obtained from the University of Lübeck’s ethics committee 19-332A.

## Results

We identified 12 patients diagnosed with BP during immune CI therapy between 2014 and 2018. Seven patients were undergoing CI for metastatic cutaneous melanoma. The remaining patients were receiving CI therapy for metastatic uveal melanoma, metastatic melanoma of unknown primary, squamous cell carcinoma of the lung, and renal cell carcinoma (**Table 1**).

**Table 1:** Clinical features of patients diagnosed with bullous pemphigoid (BP) under CI therapy.

Pat. No.	Sex/age at emergence of BP	Cancer	Treatment	Treatment response	Time interval initiation of CI therapy—BP (weeks)	Clinical symptoms of BP	Other irAEs
**1**	M/62	Amelanotic melanoma	P	CR	27	Skin: scattered papules with central vesicles Mucosa: vesicular lesions of the oral mucosa	–
**2**	M/76	Renal cell CA	N	SD	19	Skin: palmoplantar hyperkeratosis, polygonal papules and vesicles; Mucosa: vesicular and white reticular lesions of the oral mucosa	–
**3**	M/76	MM	P	PR	16	Skin: large facial bullae, minimal pruritus Mucosa: none	–
**4**	M/62	NM	P	PD	8	Skin: maculopapular erythema and bullae on the trunk Mucosa: none	–
**5**	M/78	MM of unknown primary	P	CR	74	Skin: maculopapular erythema and bullae on the trunk Mucosa: none	–
**6**	M/70	NM	N	CR	69	No information available	–
**7**	M/80	SSM	N	PR	3	Skin: bullae and pruritus affecting the trunk and extremities Mucosa: none	Maculopapular rash on the trunk
**8**	F/73	Not available	P	PR	37	Skin: single blister on the left thigh Mucosa: none	Thyroiditis de Quervain pruritus
**9**	F/76	Uveal melanoma	N + I	PD	60	Skin: pruritus, excoriations, later urticated plaques and blisters Mucosa: none	–
**10**	M/63	Squamous cell CA of the lung	N	PD	11	Skin: erythematous plaques Mucosa: none	–
**11**	M/77	MM	N	PD	55	Skin: erythematous plaques Mucosa: none	Encephalitis Grade 3
**12**	M/76	MM	N +/− Relatlimab	PD	60	Skin: erythematous plaques and one blister Mucosa: none	–

M, male; F, female; N, nivolumab; P, pembrolizumab; I, ipilimumab; CA, carcinoma, MM, malignant melanoma; NM, nodular melanoma; SSM, superficial spreading melanoma; CR, complete remission; PR, partial remission; PD, progressive disease; SD, stable disease.

**Figure 1:**
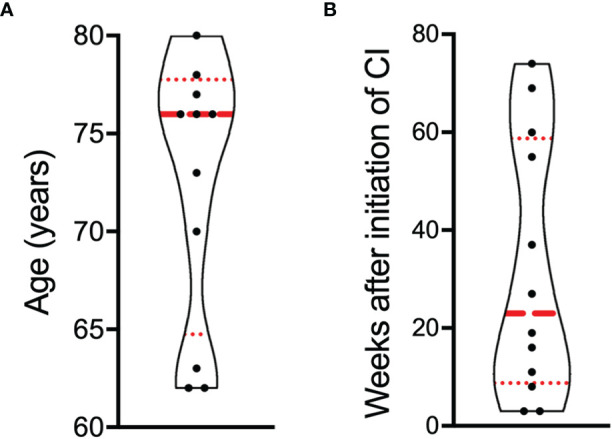
Distribution of age and latency after the initiation of CI therapy to the diagnosis of bullous pemphigoid (BP). **(A)** Age of patients at the diagnosis of BP. **(B)** Time interval in weeks between the initiation of CI therapy and the diagnosis of BP. Results are presented as violin plot. Each dot represents one patient (n = 12). The red dashed line represents the median, the red dotted lines the 25 and 75% percentiles.

Ten patients were males, two were females. The age at diagnosis of BP ranged between 62 and 80 years with a median age of 76 years (**Figure 1**). Five patients received pembrolizumab, six received nivolumab, and in one case the combination of nivolumab and ipilimumab was administered (**Table 1****)**. The median time interval from the initiation of CI therapy to the diagnosis of BP was 23 weeks and ranged between 3 and 74 weeks (**Figure 1**).

**Figure 2:**
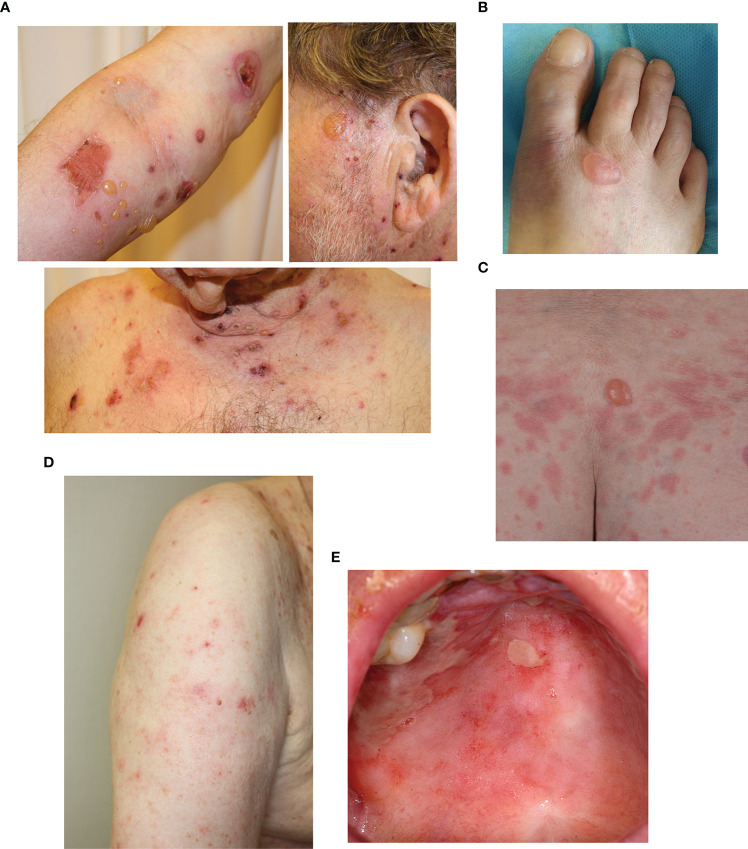
Examples of clinical manifestations of cases diagnosed as bullous pemphigoid (BP) under CI therapy. **(A)** Patient presenting with erythematous urticaria and tense blisters and erosions scattered over larger areas of the body, typical for classical BP. Patients presenting with **(B)** a single blister and **(C)** a single blister and erythematous urticaria. **(D)** Eczematous skin changes. **(E)** Oral mucosal erosions.

The clinical presentation of the 12 patients at the time of diagnosis of BP is detailed in **Table 1**, and illustrations of their clinical presentation are compiled in **Figure 2**.

Nine patients developed vesico-bullous skin changes, but in two of these patients this comprised only a single blister (**Table 1**). Two patients exhibited urticarial plaques without blistering, the typical presentation of the pre-bullous state of BP (15). Two patients additionally developed vesicles affecting the oral mucosa; the only patients to exhibit involvement of the mucous membranes.

Direct immunofluorescence (DIF) microscopy was only conducted in six of the patients. Linear IgG and C3 depositions were present in the skin biopsies from five patients, and only linear C3 deposition in one patient (**Table 2**). Indirect immunofluorescence (IIF) microscopy using monkey esophagus and/or salt-split human skin was performed in eleven patients. IgG antibodies were thereby detected in seven patients (**Table 2**). One of these patients additionally had low levels of IgA autoantibodies in the serum. Circulating IgA but not IgG antibodies, binding to the epidermal side of salt-split skin, were detected in one patient who presented with a single blister affecting the left thigh (**Tables 1** and **2**). IIF microscopy was negative in three patients (**Table 2**). The sera of nine patients were examined for anti-BP180 IgG by ELISA. Anti-BP180 IgG was detectable in four of these patients, and in another patient anti-LAD IgG was detected by immunoblot (**Table 2**). In the sera of two patients positive for anti-BP180 IgG, anti-BP230 IgG was additionally present. The histopathological findings were variable (**Table 2**), but the histopathological picture was often summarized as interface dermatitis.

**Table 2:** Summary of immuno- and histopathology.

Pat. No.	DIF	IIF	ELISA	Histopathology
**1**	n/p	IgG: +	BP180+ BP230 +	Interface dermatitis, focal epidermal necrosis
**2**	C3: +	IgG: +	BP180+ BP230 +	Orthohyperkeratosis, hypergranulosis, lichenoid interface dermatitis with a subepidermal, band-like lymphocytic infiltrate obscuring the DEJ
**3**	IgG: + C3: +	IgG: +	BP180: + BP230: −	n/p
**4**	Unspecific IgG & C3 deposition	IgG: +	BP180: − BP230: −	Subepidermal cleft, lymphohistiocytic infiltrate with single neutrophils and eosinophils
**5**	IgG: + C3: +	IgG: (+)	BP180: − BP230: −	n/p
**6**	n/p	IgG: −	BP180: − BP230: −	n/p
**7**	n/p	IgG: +	BP180: − BP230: n/p	n/p
**8**	n/p	IgA: + (DS)	BP180: n/p BP230: n/p	n/p
**9**	C3: +	IgG: + IgA: (+)	BP180: n/p BP230: n/p	Consistent with cutaneous drug reaction or BP
**10**	n/p	n/p	BP180: + BP230: n/p	Superficial interface dermatitis with eosinophilia
**11**	n/p	IgG: −	BP180: n/p BP230: n/p	Focal orthohyper- und parakeratosis, psoriasiform acanthosis, mixed inflammatory infiltrate including eosinophils
**12**	IgG: + C3: +	IgG: −	BP180: − BP230: − ^(^*^)^LAD: +	Spongiotic dermatitis, lymphohistiocytic infiltrate with eosinophils

n/p, not performed; −, negative, +, positive; +, positive; (+), faintly positive; −, negative; DS, dermal side; ^(^*^)^, assayed by immunoblot.

After the diagnosis of BP, CI therapy was permanently discontinued in four patients but switched or continued in the remaining eight patients (**Table 3**). The treatment of the skin lesions in the different centers in 11 cases included topical corticosteroids (classes II to IV according to Niedner’s classification) (**Table 3**). Three patients additionally required treatment with systemic corticosteroids, one of these patients also received three cycles of rituximab. Treatment resulted in a complete resolution of the skin findings in six patients and a partial resolution in the remaining six patients. Eleven patients remain alive, but one patient has subsequently died of metastatic melanoma.

**Table 3:** Management and outcome of bullous pemphigoid (BP).

Pat. No.	Treatment of BP	Outcome of BP	CI therapy
**1**	Topical clobetasol ointment 2x/day + prednisolone 1 mg/kg p.o. followed by a slow taper over the course of 3 months plus rituximab 375 mg/m² every 4 weeks; discontinuation after three doses due to CTCAE grade 3 thrombocytopenia	Minor alleviation	Discontinued
**2**	Palmoplantar: topical clobetasol ointment 1x/day, later mometasone ointment 1x/day; oral mucosa: triamcinolone, dexpanthenol ointment, and mouthwash	Alleviated but not completely resolved	Continued
**3**	Topical corticosteroids	Alleviated under continued topical corticosteroids but not resolved completely	Continued
**4**	Oral methylprednisolone	Resolved	Discontinued
**5**	Topical mometasone ointment	Resolved	Discontinued
**6**	Topical and systemic corticosteroids	Resolved	Switched to Pembrolizumab/discontinued
**7**	Topical corticosteroids	Ongoing	Continued
**8**	Topical prednicarbate	Resolved	Continued
**9**	Topical corticosteroids Prednisolone 15 mg/day p.o.	Resolved	Paused
**10**	Topical clobetasol ointment 2x/day	Resolved	Continued
**11**	Topical clobetasol ointment 2x/day	Ongoing	Discontinued
**12**	Topical clobetasol ointment 2x/day	Ongoing	Continued

## Discussion

We identified 12 patients who developed BP, while undergoing CI therapy for metastatic cancer, *via* a retrospective analysis of case notes at six Departments of Dermatology in Germany. Our analysis reveals that the minimal diagnostic requirements needed to confirm the presence of BP were not met in all cases, suggesting that diagnostic algorithms for BP have not been fully incorporated into routine clinical practice and testing for linear deposition of autoantibodies and/or C3 at the DEJ was not performed as standard.

In fact, linear depositions of autoantibodies or C3 at the DEJ were demonstrated in six out of twelve cases recorded as “BP during CI therapy.” It must be borne in mind that these minimal requirements do not allow a clear distinction between BP and other pemphigoid diseases such as the inflammatory variant of epidermolysis bullosa acquisita and anti-p200 pemphigoid, which cannot be clearly distinguished from BP by the clinical presentation alone. Given that linear deposition of autoantibodies at the DEJ is also present in epidermolysis bullosa acquisita and anti-p200 pemphigoid, the diagnosis of BP can only be confirmed by additionally detecting anti-BP180 IgG in the serum. This criterion was only met in four of our cases. The detection of linear depositions of IgG at the DEJ and the demonstration of anti-BP180 IgG antibodies in the serum was only met in three cases. The absence of confirmatory serum anti-BP180 IgG antibodies is a frequent finding in the hitherto reported cases of BP during CI therapy. Therefore, it is difficult to determine the true incidence and prevalence of BP during CI therapy. Nevertheless, a temporal relationship between the use of CI therapy and the development of BP and/or the development of BP during a re-challenge with CI therapy provide evidence for an irAE aetiology.

Given the complexity and clinical heterogeneity of BP as an autoimmune blistering dermatosis, let alone as an irAE, diagnostic algorithms for the accurate diagnosis of cutaneous irAEs under CI therapy are necessary not only to optimize CI therapy and improve the management of its side-effects, but also to gain new insight into the pathophysiology of autoimmune blistering diseases in general. We therefore suggest that in all cases of cutaneous irAEs under CI, including cases where severe pruritus is the only symptom, DIF microscopy for linear depositions of autoantibodies at the DEJ should be considered. If positive, serological analyses searching for autoantibodies against proteins of the dermal-epidermal adhesion complex should be conducted. The analyses should include IIF microscopy to screen for autoantibodies directed to proteins of the dermal-epidermal adhesion complex and should optimally be conducted on NaCl-split human skin because it, in contrast to the alternative substrate monkey esophagus, allows BP to be distinguished from epidermolysis bullosa acquisita and anti-p200 pemphigoid depending on where the antibodies bind. IIF microscopy should be followed by ELISA and immunoblotting analyses to precisely determine the antigen specificity of the autoantibodies. Employing this diagnostic algorithm may ensure the accurate determination of the frequency of pemphigoid diseases, possibly induced by CIs, and to pinpoint their specific features, central to improving the management of cutaneous irAEs. In fact, determination of the presence and serum concentration of autoantibodies directed to proteins of the dermal-epidermal adhesion complex could even be considered prior to the initiation of CI therapy. This may help to determine whether CI inhibition may promote the emergence of BP by facilitating the break of tolerance against proteins of the dermal-epidermal adhesion complex or by promoting the initiation of the effector phase in individuals in whom tolerance had already been broken before the administration of CIs. Ultimately, patient subgroups with increased susceptibility to BP under CI therapy could be identified to facilitate earlier recognition and treatment. Interestingly, it has recently been suggested that the development of skin autoantibodies may even be associated with improved response to CI therapy, albeit in lung cancer (16). Therefore, the detection of skin autoantibodies may actually provide additional prognostic information.

Furthermore, it is worth bearing in mind that CI therapy has also been associated with the development of lichen planus pemphigoides (LPP) (17, 18). While the clinical presentation of LPP may mimic BP, a careful correlation of the clinical, histopathological, and immunopathological features usually permits differentiation between these two conditions.

The twelve cases of BP during CI therapy reported here were mild to moderate in their clinical severity. In contrast to spontaneous BP, the skin lesions were often restricted to single body areas and treatment with topical or oral corticosteroids alone was sufficient to achieve disease control in 11 patients. Topical therapy for spontaneous BP, according to the European guidelines, usually includes whole body treatment with superpotent corticosteroids twice daily (12). However, topical therapy alone is often not sufficient to control the disease and the addition of one or two systemic treatment options, including dapsone, azathioprine, mycophenolate mofetil, doxycycline, high-dose intravenous corticosteroid pulses, intravenous immunoglobulins, or rituximab is often necessary.

It is striking that no patient required the topical treatment regimen recommended by the European guidelines and that only one patient required systemic treatment with rituximab. Although rituximab has been highlighted in a recent case report to be effective in the treatment of CI-induced BP, in our patient the effect was poor, consistent with its limited efficacy in spontaneous BP (19).

Furthermore, the histopathological analysis of lesional skin in our study, as well as in many case reports, revealed a minimal inflammatory infiltrate in the dermis. These features are reminiscent of the findings reported recently in BP induced by dipeptidyl peptidase IV inhibitors (“gliptins”) used in the treatment of diabetes (20–22), although a causal link between gliptin intake and BP has not yet been established. Similar to CI-associated disease, gliptin-associated cases tend to feature milder skin inflammation (22), and discontinuing gliptins does not lead to spontaneous reversal of disease as is the case for most other drug-induced cutaneous side-effects (20). Intriguingly, dipeptidyl peptidase IV (CD26) exerts diverse immunomodulatory roles, including regulatory functions in T cell activation similar to PD-1 and its ligands (23). It is therefore tempting to speculate that gliptins and CIs elicit BP by related molecular mechanisms.

Collectively, our retrospective analysis reveals important differences between spontaneous BP and BP-induced during CI therapy. Firstly, BP during CI therapy was milder than as is case in spontaneous BP. Secondly, the immunopathological features of spontaneous BP were only confirmed in a minority of CI-induced cases. Whilst this may result from a lack of familiarity of the diagnostic algorithms for BP in oncology, it may also reflect a milder immune response to BP180 in IC-induced BP, potentially explaining the less severe phenotype. Consequently, the immunopathological analyses usually used for the diagnosis of BP may be not sensitive enough and, with respect to ELISA assays, the cut-lines defined for BP to distinguish pathological and non-pathological autoantibody levels may be not completely applicable to diagnose CI-induced BP. In line with this latter notion, it has recently been reported that levels of anti-BP180 IgG are often increased in patients under CI therapy but in the vast majority of cases still do not reach the defined cut-off lines between the pathological and non-pathological range. Furthermore, commercially available ELISAs for the detection of anti-BP180 IgG only detect autoantibodies directed to one or both terminal ends of the NC16A domain of BP180. It cannot be excluded that blistering disease induced by CI therapy results from the production of autoantibodies against other parts of the protein. Indeed, there are also reports of CI inducing rare BP variants, including anti-LAD-1 IgG-positive, anti-BP180 NC16A IgG-negative BP (24).

Perhaps the most important question for the clinical management of CI-induced BP is whether CI therapy can be continued. Whilst the decision to continue IC therapy in patients with IC-induced BP should be made on an individual basis, carefully weighing up the risks and benefits, it is reassuring that treatment could be continued in a number of our patients. In fact, it is worth noting that similar to the case in IC-induced vitiligo (25, 26), IC-induced BP may be associated with improved treatment response and increased overall survival (27), although this remains to be confirmed in larger, prospective studies.

## Data Availability Statement

The original contributions presented in the study are included in the article/supplementary material. Further inquiries can be directed to the corresponding author.

## Ethics Statement

The studies involving human participants were reviewed and approved by Ethics Committee of the University of Lübeck.

## Author Contributions

CS, EAL, PT, RG, MF, CL, LR, FM, DG, RH, and DZ identified patients and characterized these cases. CS, EAL, and PT planned the study, analyzed the data, and wrote the paper. RG, MF, CL, LR, FM, DG, RH, and DZ revised the paper. All authors contributed to the article and approved the submitted version.

## Funding

This research was supported by DFG funding for the Clinician Research Unit 303 *Pemphigoid Diseases—Molecular Pathways and their therapeutic Potential* and for the Excellence Cluster 2167 *Precision Medicine in Chronic Inflammation*.

## Conflict of Interest

DG reports personal fees and other from Pierre Fabre Pharma Gmbh, personal fees and other from Roche Pharma AG, personal fees and other from Bristol-Myers Squibb GmbH & Co. KGaA, personal fees and other from Novartis Pharma GmbH, personal fees and other from Sanofi-Aventis GmbH, during the conduct of the study; other from Amgen GmbH, other from Janssen-Cilag GmbH, other from Galderma Laberatorium GmbH, outside the submitted work. RG reports personal fees and non-financial support from BristolMyersSquibb, personal fees and non-financial support from Roche Pharma, grants, personal fees and non-financial support from Merck Serono, grants, personal fees and non-financial support from Amgen, personal fees and non-financial support from Pierre Fabre, grants, personal fees and non-financial support from Sanofi Regeneron, personal fees from MerckSharpDohme, grants, personal fees and non-financial support from Novartis, personal fees from Almirall Hermal, grants and personal fees from Pfizer, personal fees from SUN Pharma, personal fees from 4SC, grants from Johnson&Johnson outside the submitted work. RH reports personal fees from ROCHE, personal fees from BMS, personal fees from MSD, personal fees from Novartis, personal fees from Pierre-Fabre, outside the submitted work. EL reports personal fees and non-financial support from BristolMyersSquibb, personal fees and non-financial support from Novartis, Meeting and travel support from Curevac and advisory board fees from Sun Pharma. CL reports personal fees from BMS, personal fees from MSD, personal fees from Sanofi, personal fees from Novartis, personal fees from Roche, personal fees from Pierre Fabre, personal fees from Amgen, personal fees from Kyowa Kirin, personal fees from Biontech, personal fees from Almiral Hermal, personal fees from Sun Pharma, personal fees from Merck, outside the submitted work. PT: speaker´s honoraria from BMS, Novartis, MSD, Pierre-Fabre, CureVac and Roche, consultant´s honoraria from BMS, Novartis, Pierre-Fabre, Merck Serono, Sanofi und Roche and travel support fom BMS, Pierre-Fabre and Roche.

The remaining authors declare that the research was conducted in the absence of any commercial or financial relationships that could be construed as a potential conflict of interest.
